# Prominent facilitation at beta and gamma frequency range revealed with physiological calcium concentration in adult mouse piriform cortex *in vitro*

**DOI:** 10.1371/journal.pone.0183246

**Published:** 2017-08-18

**Authors:** Marie Gleizes, Simon P. Perrier, Caroline Fonta, Lionel G. Nowak

**Affiliations:** 1 Centre de Recherche Cerveau et Cognition, Université de Toulouse, Toulouse, France; 2 Unité Mixte de Recherche 5549, Centre National de la Recherche Scientifique, Toulouse, France; University Paris Diderot, FRANCE

## Abstract

Neuronal activity is characterized by a diversity of oscillatory phenomena that are associated with multiple behavioral and cognitive processes, yet the functional consequences of these oscillations are not fully understood. Our aim was to determine whether and how these different oscillatory activities affect short-term synaptic plasticity (STP), using the olfactory system as a model. In response to odorant stimuli, the olfactory bulb displays a slow breathing rhythm as well as beta and gamma oscillations. Since the firing of olfactory bulb projecting neurons is phase-locked with beta and gamma oscillations, structures downstream from the olfactory bulb should be driven preferentially at these frequencies. We examined STP exhibited by olfactory bulb inputs in slices of adult mouse piriform cortex maintained *in vitro* in an *in vivo*-like ACSF (calcium concentration: 1.1 mM). We replaced the presynaptic neuronal firing rate by repeated electrical stimulation (frequency between 3.125 and 100 Hz) applied to the lateral olfactory tract. Our results revealed a considerable enhancement of postsynaptic response amplitude for stimulation frequencies in the beta and gamma range. A phenomenological model of STP fitted to the data suggests that the experimental results can be explained by the interplay between three mechanisms: a short-term facilitation mechanism (time constant ≈160 msec), and two short-term depression mechanisms (recovery time constants <20 msec and ≈140 msec). Increasing calcium concentration (2.2 mM) resulted in an increase in the time constant of facilitation and in a strengthening of the slowest depression mechanism. As a result, response enhancement was reduced and its peak shifted toward the low beta and alpha ranges while depression became predominant in the gamma band. Using environmental conditions corresponding to those that prevail *in vivo*, our study shows that STP in the lateral olfactory tract to layer Ia synapse allows amplification of olfactory bulb inputs at beta and gamma frequencies.

## Introduction

EEG and intracerebral LFP recordings have disclosed a variety of oscillatory phenomena in the brain. Although the precise ranges differ depending on the species and structure examined, several types of oscillations with characteristic frequencies and dynamic features have been associated with different sleep/waking states and with various perceptual and cognitive processes. For example, non-REM sleep is characterized by the presence of a slow sleep rhythm (< 1Hz), of delta oscillations (1–4 Hz) and short epochs of sleep spindles (11–15 Hz) [1–3]. Theta waves (typically 6–12 Hz in awake rodents) have been mostly studied in the hippocampus where they occur during a variety of behaviors as well as during REM sleep [4, 5]. Alpha waves (8–12 Hz), most prominent in visual cortex, are associated with rest [6] and may be involved in awareness and attention [7]. Beta waves (between 12 and 25–35 Hz) are most salient in sensorimotor cortex [8–10] and may also be involved in attentional processes [11, 12]. Gamma oscillations, with frequencies larger than 25–35 Hz, are typically observed during the processing of sensory stimuli in visual [13–16], somesthetic [17, 18] and auditory cortices [19, 20] as well as during various cognitive tasks involving awareness, attention and memory (reviewed in: [21, 22]).

Although they are clearly associated with a variety of perceptual and cognitive processes, the functional significance of oscillations, in particular beta and gamma oscillations, has remained elusive. At one extreme, it has been proposed that oscillations are merely an epiphenomenon of the functioning of cerebral networks (e. g., [23–25]) while at the other, it has been proposed that oscillations play prominent roles in feature binding, awareness and/or conscious perception (e. g., [22, 26–28]).

Here we adopted a “bottom-up” approach to try to learn more on the functional impacts of oscillations. More precisely, we examined the *consequences* of oscillatory activity on synaptic transmission. Indeed, synaptic transmission is not steady: on a short time range (e. g., less than a few minutes) postsynaptic responses usually display reversible dynamic changes, referred to as short-term plasticity (STP). Synaptic efficacy can thus be modulated by two broad types of STP: short-term facilitation (STF) and short-term depression (STD) (e. g., [29]). Several STF and STD mechanisms have been disclosed, each characterized by different time courses, which range from a few milliseconds up to hundreds of seconds, and may coexist in the same synapse. Thus, under the proviso that presynaptic action potentials are phase-locked with oscillations, we expected features of STP in postsynaptic neurons to change depending on the oscillatory frequency in the presynaptic structures and neurons.

We examined STP in the piriform cortex, one of the main relays of olfactory information after integration in the olfactory bulb (e. g., [30–32]). Three types of oscillations have been identified in the olfactory bulb. Breathing itself induces oscillations, which in rodents display frequencies between 1 and 5 Hz at rest and between 6 and 10 Hz during active sniffing (e. g., [33–35]). Presentation of odorant stimuli further induces both beta (between 15 and 35–40 Hz) [33, 36–43] and gamma (>35–40 Hz) fluctuations in the LFPs [33, 35, 36, 38–49]. Importantly, the firing of both mitral and tufted cells, the two projecting cell types of the olfactory bulb, appears to be phase-locked to both beta [40, 42] and gamma oscillations [40–42, 48–50]. Thus in physiological conditions, synapses in layer Ia of the piriform cortex, where terminals of olfactory bulb efferent neurons are concentrated (e. g., [30, 51, 52]), should be activated at frequencies corresponding to those observed in the olfactory bulb. Here we examined STP in layer Ia of the piriform cortex *in vitro*, while applying electrical stimulation in the lateral olfactory tract (LOT), which contains olfactory bulb efferent axons. The stimulation frequency range (0.1 to 100 Hz) encompasses that observed in natural brain rhythms.

Three conditions had to be met to obtain a relevant description of the effect of different stimulus frequencies on STP *in vitro*: 1) use of several stimuli in a train in order to be able to observe the dynamics of STP, rather than the classical stimulus pair that limits the analysis to paired-pulse ratios; 2) model based, quantitative analysis of the data to obtain relevant parameter values for mechanisms of STD and STF, to be able to go beyond the mere phenomenological description of changes in response amplitude; 3) use of adult animals and of an extracellular medium that most closely mimics the one encountered in the adult brain *in vivo*; in particular, most *in vitro* experiments use an extracellular concentration of calcium of 2–2.5 mM while that measured *in vivo* is of the order of 1 mM; given the importance of calcium in synaptic transmission, this factor-two difference is likely to strongly affect STP and its dependence on stimulation frequency. Although 1 or 2 of these conditions have been met in the numerous studies devoted to STP in piriform cortex, our study would be the first where all 3 requirements were fulfilled: our experiments were performed using brain slices issued from adult mice and were maintained in an *in vivo*-like ACSF; we used stimulation trains of 5 stimuli and extracted the parameters of STP by fitting the data with a model derived from that initially developed by Tsodyks and Markram (1997) [53]. Results were compared with those obtained with a “classical” calcium concentration (2.2 mM).

Our experimental results and model parameters show that the interplay of STF and STD resulted either in an increase or a decrease of response amplitude that depended on stimulus frequency and calcium concentration. In the presence of high calcium concentration (2.2 mM), synaptic responses were enhanced, though weakly, at stimulation frequencies corresponding to the low beta and alpha bands while depression tended to occur at stimulation frequencies within the gamma band. On the other hand, in the presence of an *in vivo*-like calcium concentration (1.1 mM), response enhancement displayed a larger dynamic range. Additionally, response enhancement was maximal in the beta range and was also strong in the gamma range. These results suggest that short-term plasticity in layer Ia of the piriform cortex allows amplification of synaptic responses at frequencies corresponding to odor-induced beta and gamma oscillations in the olfactory bulb.

## Material and methods

### Ethics statement

All procedures were conducted in accordance with the guidelines from the French Ministry of Agriculture (décret 87/848) and from the European Community (directive 86/609) and was approved by the local ethical committee (comité d’éthique Midi-Pyrénées pour l’expérimentation animale, N° MP/06/79/11/12).

### Slices preparation

The protocol for brain slice preparation was adapted from those previously described [54, 55] and is briefly summarized here. Two- to 4-month-old C57BL/6 female mice were used for these experiments. Mice were deeply anesthetized with isoflurane and then rapidly decapitated. The brain was removed and prepared for slicing in ice-cold, oxygenated (95% O_2_ / 5% CO_2_) modified artificial cerebrospinal fluid (mACSF) of the following composition (mM): NaCl 124, NaHCO_3_ 26, KCl 3.2, MgSO_4_ 1, NaH_2_PO_4_ 0.5, MgCl_2_ 9, Glucose 10. Note that Ca^++^ was omitted while the final Mg^++^ concentration was 10 mM. Studies showed that axons innervating the piriform cortex leave the LOT at right angle [56], yet the lateral olfactory tract is not parallel to the long axis of the brain. Therefore, in order to preserve axons at best, a cut was made through the brain in the frontal plane with an angle of 70° at the level where cortex and cerebellum are adjacent. The brain was glued on a pedestal by the posterior side, the apex of the brain facing upward. Four hundred-micrometer-thick slices were then cut on a vibratome in the presence of cold, oxygenated mACSF. Slices were allowed to recover for at least 1 hour at room temperature in a holding chamber filled with oxygenated *in vivo*-like ACSF.

### *In vivo*-like ACSF composition

Synaptic transmission and STP are highly sensitive to extracellular ion concentrations, in particular that of calcium. We therefore used extracellular ionic concentrations close to those observed *in vivo*. Extracellular K^+^ and Ca^++^ concentrations in the interstitial fluid have been reported in numerous studies using ion selective microelectrodes. Using the available literature for rodents [57–70], we calculated the median concentrations for K^+^ and Ca^++^ and obtained values of 3.2 mM and 1.1 mM, respectively. Mg^++^ concentration is of importance too as Mg^++^ may partially antagonize voltage-dependent calcium channels. Mg^++^ concentration is less documented than that of K^+^ and Ca^++^ but values for concentration in the CSF of the mouse [71] and of other species (e. g., [72–76]) appear to be about to 1 mM. We also used a phosphate concentration of 0.5 mM, similar to that measured in the CSF [74, 77, 78]. Our *in vivo*-like ACSF was therefore composed of (in mM): NaCl 124, NaHCO_3_ 26, KCl 3.2, MgSO_4_ 1, NaH_2_PO_4_ 0.5, CaCl_2_ 1.1, and glucose 10. This ACSF was continuously bubbled with a 95% O_2_ / 5% CO_2_ mixture (pH 7.4).

### Stimulation and recording

For recording, an individual slice was transferred in a submersion type chamber that was continuously gravity fed with oxygenated *in vivo*-like ACSF at a flow rate of 3–3.5 ml/min. All recordings were performed at 34–35°C. Local field potentials were recorded in layer Ia of the piriform cortex either through tungsten-in-epoxylite microelectrodes (FHC, 0.2–0.3 MΩ) or through glass micropipettes filled with ACSF (3–7 MΩ). When recorded through microelectrodes, the signal was amplified (x1000) and filtered (0.1 Hz-10 kHz) with a NeuroLog system (Digitimer, UK). When recorded through micropipettes, the signal was first amplified on an AxoClamp 2B amplifier (Axon Instrument, Foster City, CA), further amplified with a Neurolog post-amplifier (final gain: ×1000) and low-pass filtered at 10 kHz.

Intracellular recording were performed in layer II of the piriform cortex using sharp micropipettes filled with 3 M K-Acetate (50–90 MΩ). Intracellular signal was amplified with the AxoClamp 2B amplifier (gain ×10). Criteria for accepting intracellular recording data were: stable membrane potential more negative than -60 mV, input resistance > 20 MΩ and ability of the cell to repetitively fire overshooting action potentials during depolarizing square current pulses lasting 120–300 msec.

Micropipettes for LFP or intracellular recordings were pulled on a P97 Flaming Brown micropipette puller from 1.2 mm OD medium walled capillaries with filament (GC120F, Harvard Apparatus).

Fifty Hz noise was eliminated with a Humbug system (Quest Scientific, Canada). All signals were digitized with a digitization rate of 20–50 kHz (1401plus or power1401, CED, UK). Real-time display of the signals was achieved with an oscilloscope and with Spike2 software (CED, UK).

Extracellular electrical stimulation was applied through tungsten-in-epoxylite microelectrodes (FHC, 0.2–0.3 MΩ) implanted in the LOT and consisted in monopolar cathodal square current pulses (6–35 μA, 200 μs duration) delivered by an isolated stimulator (A365 stimulus Isolator, WPI).

The LFP recorded in layer Ia was composed of a fiber volley followed by a slow negative wave (N-wave, [79]). The fiber volley corresponds to the summation of synchronous action potentials traveling in axons while the N-wave reflects the summation of excitatory postsynaptic potentials generated in the vicinity of the recording electrode [51]. Stimulation intensity was kept in a range allowing reliable N-wave generation but low enough to avoid contamination by fast positive components that presumably resulted from postsynaptic action potential generation [80]. Low intensity also limited effective current spread to a few tens of μm [54], hence to within the LOT. Response amplitude was measured as the maximal amplitude of the N-wave relative to prestimulus baseline. The amplitude of the fiber volley was measured similarly.

In order to examine the effect of Ca^++^ on synaptic response amplitude, 6 concentrations of Ca^++^ have been used (0.3, 0.5, 0.7, 0.9, 2.2 and 3.3 mM) in addition to the 1.1 mM control concentration. The time course of the effect of modifying calcium concentration on field potential amplitude was monitored using electrical stimulation delivered at 0.5 Hz for 15–20 minutes. One or two calcium concentrations were tested between one control and one recovery test; data were included only if the value during recovery differed by less than 15% from that obtained in the control period.

STP has been examined in 1.1 and 2.2 mM Ca^++^. When switching from one calcium concentration to another, ten minutes of stable baseline was required before proceeding to the STP protocol. In each experiment, STP was tested using stimulation trains consisting of five consecutive stimuli delivered at 6 different frequencies: 3.125, 6.25, 12.5, 25, 50 and 100 Hz. Each train was repeated 10 times to allow for averaging. The trains were spaced apart by ten seconds without stimulation. The number of stimuli was limited to 5 per train for two reasons: first because the number of successive oscillation cycles in the olfactory bulb is typically between 4 and 10 (e. g., [33, 38, 42, 48]), and second, because large number of pulses may recruit a slow adaptation (see Discussion) that would have hampered our model-based analysis.

### Data analysis

Signal processing was performed using the Spike2 software with scripts written by the users. For the analysis of the effect of calcium on synaptic transmission at 0.5 Hz, signals were averaged over the last minute (thirty responses) of the 15–20 min-long series of stimuli. Amplitudes obtained in a given calcium concentration were normalized by that obtained in control (1.1 mM) calcium concentration (*normalized response amplitude*, NRA).

For STP analysis, the 10 traces obtained at a given frequency and with the same ordinal stimulus number were averaged. The individual-level data are represented as the mean ± SEM. For population data analysis and for STP model fitting, we first calculated the *relative response amplitude* (*RA*_*n*_) by dividing the amplitude of the N-wave obtained at the *n*^th^ stimulation (*A*_*n*_*)* by that obtained at the first stimulation (*A*_*1*_) in each stimulation train (*RA*_*n*_ = *A*_*n*_ / *A*_*1*_, *n* between 1 and 5). Note that data obtained in 2.2 mM calcium were normalized by the first response amplitude in the *control* (1.1 mM) condition for each frequency.

### Short-term plasticity model

We developed a phenomenological model to compute the parameters of short-term synaptic plasticity at the LOT-layer Ia synapse. The model, implemented in *R* (R-project v.3.3.1), is derived from models described in previous studies [53, 81, 82]. It included one term of facilitation and two terms of depression.

In response to the first stimulation of a train, the relative response amplitude is:

$$RA_{1}=E\times U=1$$

where *E* represents the maximal synaptic efficacy and *U*, the utilization of efficacy *E*, represents the fraction of *E* that is used at the first stimulation. *U* may be envisioned as the increase in calcium concentration in the presynaptic terminals that leads to neurotransmitter release, while *E* would correspond to the theoretical maximal value obtained if the synapses released all their synaptic vesicles and/or if all postsynaptic receptors saturated.

The purpose of the model was then to approximate, for each stimulation rank *n* >1, *RA* as the product of four terms:

$$RA_{n}=E\times r_{1,n}^{-}\times r_{2,n}^{-}\times u_{n}^{+}$$

where *'minus'* designates values just before the stimulation and *'plus'* designates the value at stimulation time. *u*, the utilization of efficacy, varies so as to implement facilitation: at each stimulation, *u* rises by a fraction of the parameter *U*; then, during the interpulse interval (*IPI*), *u* decays to zero according to the time constant of facilitation (*τ*_*F*_) as follows:

$$\mathrm{at}\;\mathrm{stimulation}\;\mathrm{time}:\;u_{n+1}^{+}=u_{n+1}^{-}+U\times (1-u_{n+1}^{-});$$

$$\mathrm{during}\;IPI:\;u_{n+1}^{-}=u_{n}^{+}\times e^{\frac{-IPI}{\tau_{F}}}.$$

*r*_***1***_ and *r*_***2***_ designate two available reserves of *E*, that can be assimilated to synaptic resources such as vesicles of neurotransmitters or availability of postsynaptic receptors. At rest, *r*_***1***_ = *r*_***2***_ = 1. At each stimulation, both reserves are decremented in proportion to the utilization of efficacy, *u*. Furthermore, *u* is assumed to be shared between both synaptic reserves by factors *k* and (1-*k*), respectively. During the *IPI*, *r*_*1*_ and *r*_*2*_ recover with time constants that correspond to two time constants of recovery from depression (respectively, *τ*_*R1*_ and *τ*_*R2*_), as follows:

$$\mathrm{at}\;\mathrm{stimulation}\;\mathrm{time}:\;r_{1,n+1}^{+}=r_{1,n}^{-}-r_{1,n}^{-}\times u_{n+1}^{+}\times k,\;r_{2,n+1}^{+}=r_{2,n}^{-}-r_{2,n}^{-}\times u_{n+1}^{+}\times (1-k);$$

$$\mathrm{during}\;IPI:\;r_{1,n+1}^{-}=(r_{1,n}^{+}-1)\times e^{\frac{-IPI}{\tau_{R1}}}+1,\;r_{2,n+1}^{-}=(r_{2,n}^{+}-1)\times e^{\frac{-IPI}{\tau_{R2}}}+1.$$

For each sample, including either one or two calcium concentrations and five or six stimulation frequencies, the model was fit to the data using an iterative procedure that minimized the mean-squared error (MSE) between the recorded *RA* and the amplitudes predicted by the model. The method to determine MSE was an implementation of that of Nelder and Mead (1965) [83], that uses only function values and is robust although relatively slow. *E* was assumed to be independent of extracellular calcium concentration and therefore one single value of *E* was determined in the paired calcium manipulation experiments. One single set of parameters was determined for an experimental block containing one (6 parameters) or both (11 parameters) calcium concentration condition. During parameters optimization, *E*, *U*, *τ*_*F*_, *τ*_*R1*_, *τ*_*R2*_ and *k* were constrained as follows: *E*, *U* and *k* between 0 and, respectively, 10, 1 and 1; time constants between 0 and 3 seconds with the supplementary constraint that *τ*_*R1*_ had to be inferior to *τ*_*R2*_. When optimal *k* was equal to 1, *τ*_*R2*_ had no more influence and was withheld from further analysis. Robustness of fitting was estimated using the root mean-squared error (RMSE). For the present data set, the RMSE ranged between 0.017 and 0.113.

### Statistics

ANOVA and Fischer's PLSD posthoc tests were used to examine the effects of calcium concentration and stimulation frequency on response amplitude. Paired t-test was used to compare model parameter values in 1.1 and 2.2 mM Ca^++^. Unless otherwise stated, population data are summarized by their means and 95% confidence intervals; values between brackets in text correspond to the 95% confidence interval. Unless otherwise stated, error bars in Figures delimit the 95% confidence interval.

## Results

### Effect of extracellular calcium concentration on response amplitude

We first examined the effect of varying the extracellular calcium concentration on the amplitude of the synaptic responses at the LOT-layer Ia synapse of piriform cortex using a stimulation frequency of 0.5 Hz. This frequency induced a very weak depression (-3% between first pulse response amplitude and steady state on average) but response amplitudes were measured only once the steady state was reached. Effect of varying extracellular calcium concentration has been examined in 16 experiments. Six extracellular calcium concentrations were tested in addition to the control concentration at 1.1 mM (n = 18): 0.3 (n = 3), 0.5 (n = 5), 0.7 (n = 4), 0.9 (n = 4), 2.2 (n = 8) and 3.3 mM (n = 2). One to four different concentrations were tested in one experiment.

**Fig 1:**
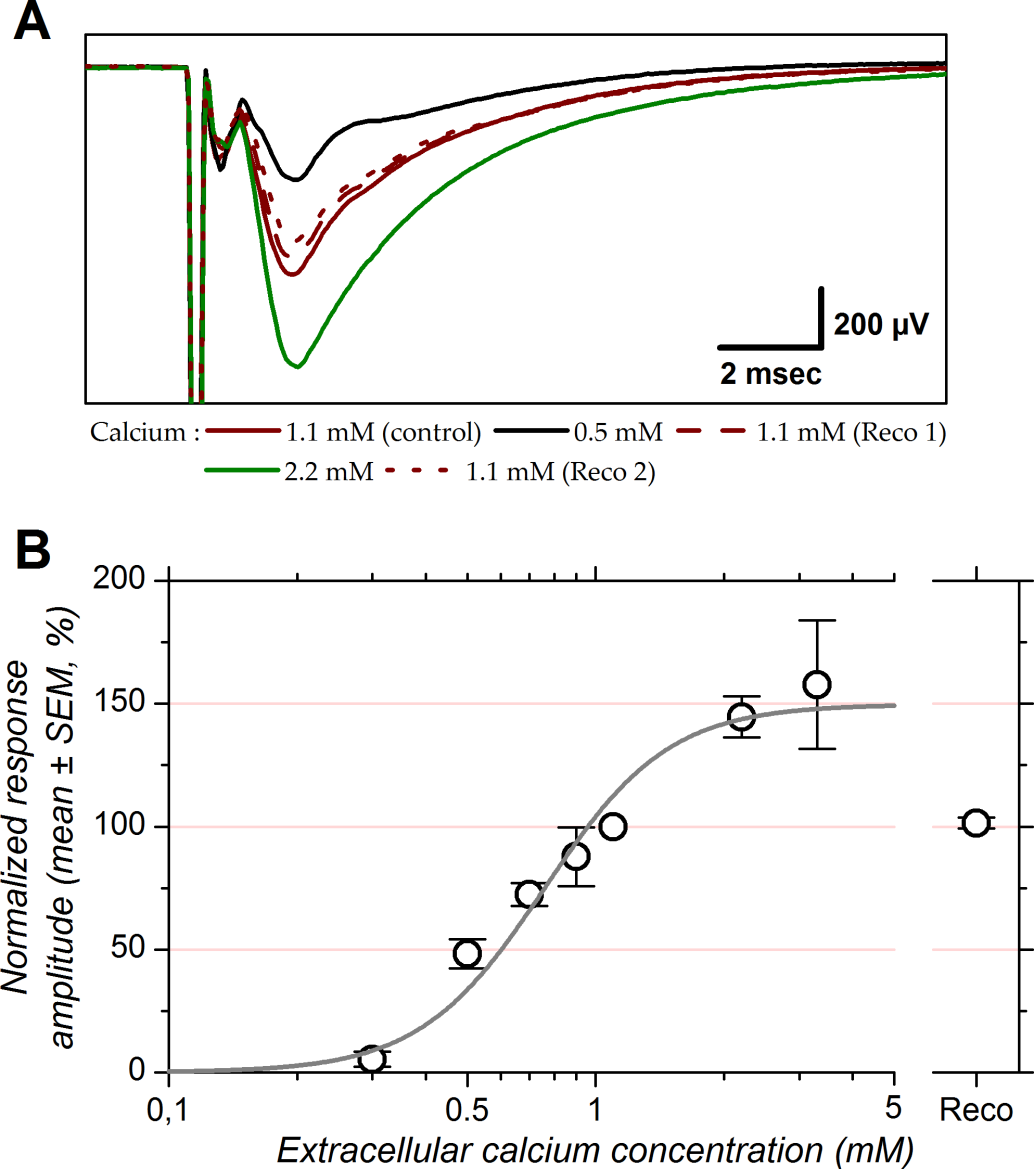
Effect of extracellular calcium concentration on the amplitude of the response evoked at 0.5 Hz at the LOT-layer Ia synapse. *A*: LFP recorded in layer Ia of the piriform cortex with 3 different extracellular calcium concentrations (0.5, 1.1 and 2.2 mM). Stimulation intensity was 15 μA. *B* shows Hill equation fitted to population data. Data points correspond to the mean and error bars to ± 1 SEM computed after normalizing the individual amplitudes to their corresponding control values in 1.1 mM calcium (expressed as a percentage). Continuous line corresponds to Hill equation fitted to the data (R^2^ = 0.98). “Reco”: recovery, mean normalized response amplitude upon return to 1.1 mM calcium.

Fig 1A shows an example of LFP recorded in layer Ia with 3 different extracellular calcium concentrations. In the presence of 0.5 mM calcium, the amplitude of the N-wave was about twice smaller than in the control condition, while it was approximately twice larger with 2.2 mM calcium. Control and recoveries displayed similar amplitudes. The amplitude of the fiber volley did not change, indicating that changes in N-wave amplitude were of synaptic origin and did not result from changes in axonal excitability. At the population level, fiber volley amplitude was not significantly affected by changes in calcium concentration (ANOVA, *P* = 0.07, not illustrated). In contrast, N-wave amplitude depended strongly on calcium concentration (ANOVA, *P* < 0.0001).

Population data for the N-wave are presented in Fig 1B. Before averaging, data were normalized: the normalized response amplitudes, *NRA*, correspond to the peak amplitudes of the N-wave in a given Ca^++^ concentration expressed as a percentage of the response amplitude in the control condition (1.1 mM calcium). The mean *NRA* as a function of extracellular calcium concentration, *C*, was fitted with Hill function:

$$NRA=A_{\max}\times \frac{C^{h}}{C^{h}+C_{0.5}{}^{h}}$$

with *A*_*max*_ representing the maximal response amplitude, *C*_*0*.*5*_ the extracellular calcium concentration eliciting half of *A*_*max*_ and *h* the Hill coefficient. Synaptic responses were virtually suppressed (8% of control) with a calcium concentration of 0.3 mM. Reducing calcium concentration from 1.1 mM (control) to 0.5 mM reduced response amplitude to approximately half (48%), yet doubling calcium concentration from 1.1 mM to 2.2 mM did not double response amplitude. Instead, response amplitude was increased by +45% relative to control. This indicates the presence of a saturation level which is reflected by the *A*_*max*_ of the fit at +50 ± 10 (SE) %. Relative to *A*_*max*_, the *C*_*0*.*5*_ has a value of 0.76 ± 0.05 (SE) mM. The Hill coefficient returned by the fit was 2.98 ± 0.33 (SE). The Hill coefficient suggests that the calcium sensor responsible for neurotransmitter release is activated by the binding of 3 calcium ions. These results suggest that the effect of extracellular calcium on response amplitude saturates when its bath concentration is larger than 3 mM and indicate that response amplitude in 2.2 mM calcium is already close to saturation.

### Effect of calcium on short-term plasticity

**Fig 2:**
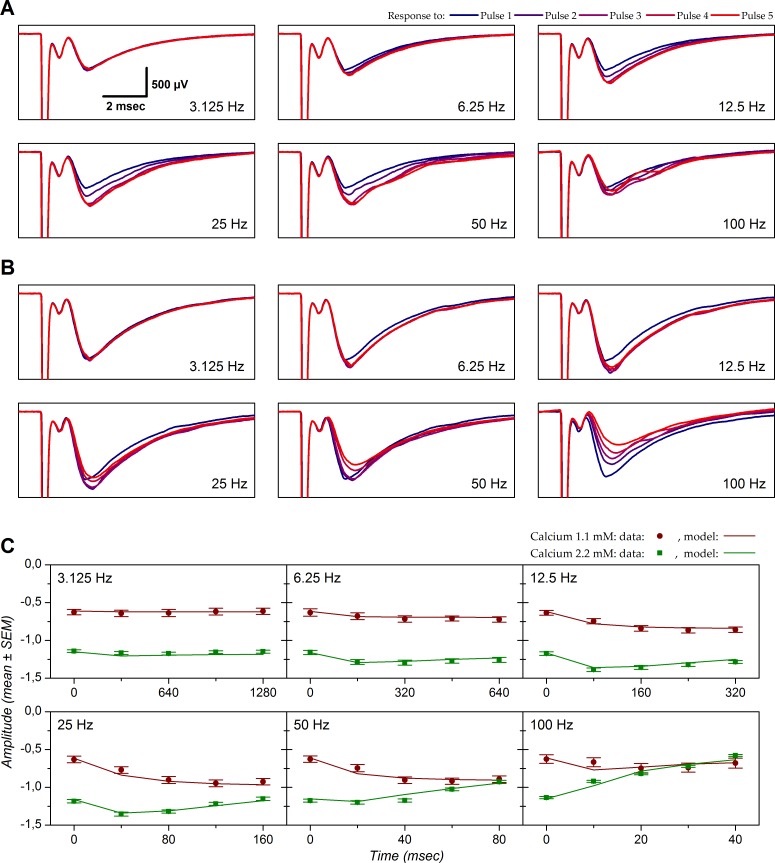
Short-term plasticity with 1.1 mM or 2.2 mM extracellular calcium in one experiment. LFPs evoked in layer Ia by stimuli applied in the LOT. Stimulation intensity was 20 μA. *A*: Response obtained with an extracellular calcium concentration of 1.1 mM. Each panel corresponds to one stimulation frequency. Each panel shows five traces that correspond to the five stimuli of the train (rank color code on top of figure). Each trace corresponds to the average of 10 sweeps. *B*: Same as *A* but in 2.2 mM calcium. Scale presented in the 3.125 Hz panel in *A* applies to all the other panels in *A* and *B*. *C*: N-wave amplitude represented as a function of stimulus time. The data points correspond to the experimental values (mean ± SEM) and the lines correspond to the result of the model fitted to the data. Model parameters were optimized for both conditions (1.1 mM and 2.2 mM) at once. Parameter *E* was shared for both conditions. Parameters values in 1.1 mM Ca^++^: *U* = 0.353, *τ*_*F*_ = 92 ms, *τ*_*R1*_ = 18 ms, *τ*_*R2*_ = 87 ms, *k* = 0.980. Parameters values in 2.2 mM Ca^++^: *U* = 0.666, *τ*_*F*_ = 223 ms, *τ*_*R1*_ = 15 ms, *τ*_*R2*_ = 418 ms, *k* = 0.909. Shared parameter *E* = 2.761. MSE value associated with this fit: 0.003. For illustration purpose the predicted RA has been de-normalized (RA multiplied by response amplitude obtained with first stimulus) to be presented on the same scale as the experimental data.

STP was tested with trains of five stimulating pulses delivered at frequencies between 3.125 and 100 Hz. Effects of different stimulation frequencies on the postsynaptic responses are exemplified in Fig 2. Fig 2A displays the LFP traces for each of the 5 successive stimuli of the trains for each frequency tested in the presence of 1.1 mM extracellular calcium. Increasing stimulation frequency did not modify fiber volley amplitude (Fig 2A). This constancy implies that the changes in N-wave amplitude were determined by changes at the level of synaptic transmission. The amplitude of the N-wave as a function of stimulus time for the different frequencies tested is represented by the data points in Fig 2C. The successive responses evoked by the 3.125 Hz train displayed identical amplitudes. An enhancement of N-wave amplitude during the stimulation train became visible at 6.25 Hz. This enhancement was maximal at 25 Hz; at this frequency the amplitude of the N-wave induced by the fourth stimulus of the train was 1.5 time larger than that induced by the first. Amplitudes were only marginally smaller with the third and fifth stimulus of the same train. At 50 Hz, response amplitude reached a maximum with the third stimulus that was slightly smaller than that obtained at 25 Hz (× 1.45 relative to first stimulus); response amplitude for the fourth and fifth stimulus plateaued at comparable levels. At 100 Hz response enhancement was barely visible (× 1.2 for the third stimulus).

In addition to increasing N-wave amplitude, increasing extracellular calcium concentration to 2.2 mM also modified the features of STP (Fig 2B and 2C). Although less marked than with 1.1 mM calcium, response enhancement occurred with stimulation frequencies in the range 3.125–25 Hz. Yet at 12.5 and 25 Hz, maximal enhancement was visible with the second stimulus of the train and response amplitude obtained with the following stimuli declined back toward the amplitude obtained with the first stimulation. At 50 Hz and, more dramatically, at 100 Hz, response enhancement was no longer observed and was instead replaced by a progressive reduction of response amplitude that was not observed in 1.1 mM calcium. In the end, response amplitude obtained with these frequencies declined toward the values observed with 1.1 mM calcium, with the 5^th^ stimulation at 50 Hz and with the 3^rd^ one at 100 Hz (Fig 2C).

**Fig 3:**
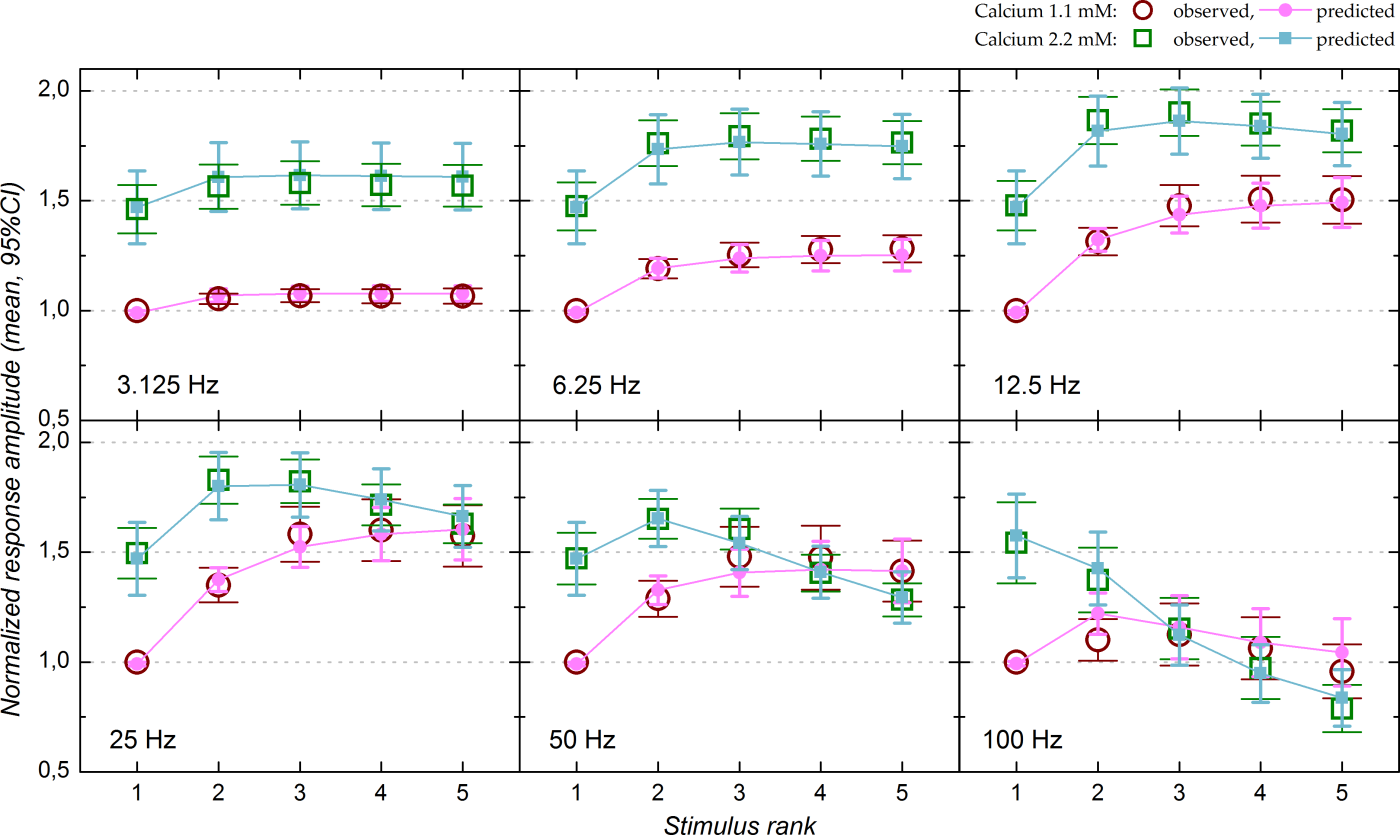
Short-term plasticity in 1.1 and 2.2 mM calcium: Population data (n = 17 experiments with 1.1 mM calcium and 8 experiments with 2.2 mM calcium). Before averaging, N-wave amplitudes in both 1.1 and 2.2 mM calcium were normalized by that obtained with the first stimulus of each trains in 1.1 mM calcium. Error bars correspond to the 95% CI. Stimulation ranks are presented on the x-axis. Hollow red circles and green squares represent the means of the experimental data in 1.1 and 2.2 mM calcium, respectively. Filled magenta circles and blue squares correspond to the means of the values predicted by the model in 1.1 and 2.2 mM calcium, respectively.

The example in Fig 2 is representative of what we observed at the population level (Fig 3). Prior to averaging, response amplitudes in both 1.1 and 2.2 mM calcium were normalized by the amplitude obtained with the first stimulation of each stimulus trains in 1.1 mM calcium. Fiber volley amplitude was not modified by stimulus frequency (ANOVA, *P* = 0.70 in 1.1 mM calcium, *P* = 0.99 in 2.2 mM calcium). In contrast, N-wave response amplitude was strongly and significantly depended on stimulation frequency (*P*<0.0001 in both 1.1 and 2.2 mM calcium) as well as on pulse ordinal number (*P*<0.0001 in both 1.1 and 2.2 mM calcium).

Population data with 1.1 mM extracellular calcium (n = 17) are represented by open red circles in Fig 3. Response enhancement was weak at 3.125 Hz (+7% relative to first pulse response). Although weak, the increase in response amplitude was significant for pulses 2–5 in comparison to pulse 1 (Fisher's PLSD, *P*<0.0001). Response enhancement was slightly stronger (+28%) at 6.25 Hz and quite important at 12.5 Hz (+50%). The maximal enhancement was achieved at 25 Hz (+60%). At 50 Hz the response enhancement (+48%) was less marked than at 25 Hz. For these four frequencies, the maximal enhancement was reached with the third pulse of the stimulus train (pulse 1 vs. pulse 3: *P*<0.0001 at 6.25–50 Hz; pulse 2 vs. pulse 3: *P* = 0.001 at 6.25 Hz, *P*<0.0001 at 12.5 and 25 Hz, *P* = 0.0001 at 50 Hz) while amplitude for the third, fourth and fifth pulses did not differ significantly, indicating that response amplitude reached a plateau level at the third pulse. Response enhancement (+12% max) was largely lost at 100 Hz: response amplitude with the second and third pulses were significantly larger than with first pulse (*P* = 0.03 and 0.008 respectively) but those with the fourth and fifth pulse were not significantly different from that with the first pulse (*P* = 0.2 and 0.3 respectively).

In 2.2 mM calcium (n = 8; open green squares in Fig 3) the amplitude of the response evoked by the first stimulus in each train was 49% higher on average than in 1.1 mM calcium, as shown above (Fig 1). As in 1.1 mM calcium, a weak response enhancement was observed at 3.125 Hz (maximum +8% relative to first pulse response amplitude) as well as a slightly stronger enhancement at 6.25 Hz (maximum +22%). For both frequencies, response amplitude reached a plateau at the second stimulating pulse (pulse 1 vs. pulses 2–5: *P*<0.0001 but no significant difference between pulses 2–5). For higher stimulation frequencies, the dynamics of STP diverged from that observed in 1.1 mM calcium: first, maximal response enhancement was obtained at 12.5 Hz (+29%) rather than at 25 Hz (+22%). Second, in 2.2 mM calcium responses at 12.5 Hz and 25 Hz were not followed by a plateau but by a decline in response amplitude: response amplitude was significantly less for the fifth pulse compared to the third one at 12.5 Hz (*P* = 0.03), and significantly less for the fourth and fifth pulse compared to the third one at 25 Hz (*P* = 0.02 and 0.0001, respectively). Despite this decrement, response amplitudes with the fifth stimulus were still larger than in 1.1 mM calcium (12.5 Hz: *P*<0.0001; 25 Hz: *P* = 0.005).

At 50 Hz the trends observed at 25 Hz were accentuated: response enhancement was weaker (maximum +12% with pulse 2), although still significant (*P* = 0.001 and 0.01 for second and third pulse responses compared to first pulse response), and was followed by a stronger response decline, such that response amplitude with the fifth pulse was actually less (-13%) than with the first (*P* = 0.0007). This decline brought response amplitude back to the values obtained in 1.1 mM calcium for the fourth and fifth stimulation pulse (*P* = 0.4 and 0.9 respectively). At 100 Hz there was no response enhancement in 2.2 mM calcium and response amplitude instead declined progressively throughout the stimulation train (-49% between the first and fifth pulse). All pulse comparisons were significant (*P* between 0.0004 and <0.0001). This strong decline brought response amplitude to values comparable to those obtained in 1.1 mM calcium at the third pulse and response amplitude for the third, fourth and fifth stimulation did not differ significantly in 1.1 and 2.2 mM calcium (P = 0.2, 0.9 and 0.3 respectively).

Altogether, although response amplitude for the first stimulation was higher in 2.2 mM calcium, weaker facilitation and/or stronger depression progressively attenuated this difference, or even suppressed it at high stimulation frequency.

**Fig 4:**
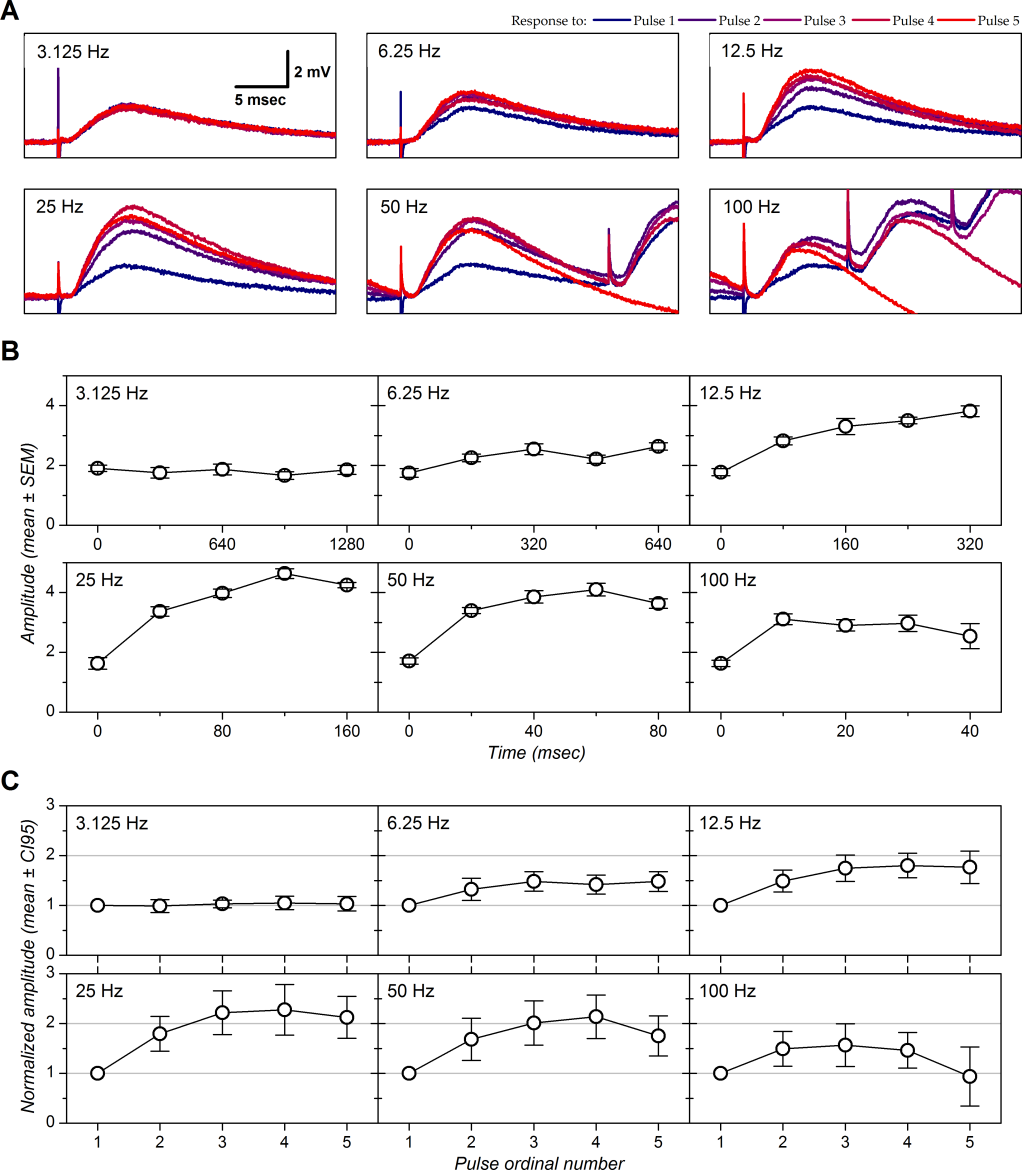
Short-term plasticity in intracellularly recorded cells with 1.1 mM extracellular calcium. *A*: Postsynaptic responses evoked by electrical stimulation applied in the LOT (stimulation intensity 6 μA). Each panel corresponds to one stimulation frequency. Each panel shows five traces that correspond to the 5 stimulus ranks of the train (color code on top of figure). Each trace correspond to the average of 8 sweeps. *B*: EPSP amplitude as a function of stimulus time. Amplitudes were measured at a delay of 6 msec after stimulation and were calculated relative to baseline measured at the foot of each EPSP in order to cancel effect of temporal summation at high frequency. The data points correspond to the experimental values (mean ± SEM). *C*: Population data (n = 5). EPSP amplitudes were normalized before averaging by the amplitude obtained with the first stimulus of each trains. Error bars correspond to the 95% CI.

We also performed intracellular recordings in order to more precisely identify the synaptic responses evoked by LOT stimulation and their STP. Intracellular recordings were performed in layer 2 of piriform cortex. We concentrated on 5 cells with monosynaptic excitatory postsynaptic potentials. The mean resting membrane potential was –79 mV [–79 - –85] and the mean input resistance was 38 MΩ [27–43]. In response to suprathreshold depolarizing current pulses, the cells emitted wide action potentials (half width at half-height: 0.64 msec [0.61–0.67]) and spike firing was regular with adaptation (not illustrated). These features suggest these cells were semi-lunar cells [84]. Effect of stimulation frequency on postsynaptic response is illustrated by one example in Fig 4A and 4B; the population data are presented in Fig 4C. Extracellular calcium concentration was 1.1 mM in all cases. Qualitatively, response amplitude as a function of stimulation frequency and of stimulus ordinal number showed the same features as observed with extracellularly recorded N-wave in 1.1 mM calcium: response amplitude showed little change when elicited at 3.125 Hz. Response enhancement was observed with higher stimulation frequencies and showed a maximum at 25 Hz. At 6.25–25 Hz, response amplitude reached a plateau at the third stimulation. Enhancement was only weak at 100 Hz and the response amplitude at this frequency returned to control response amplitude with the fifth pulse of the train. No synaptic summation was observed at 25 Hz; this suggest that response enhancement, that was maximal at this frequency as for the N-wave, was not due to nonlinearities introduced by postsynaptic membrane potential changes, such as recruitment of NMDA receptors [85] or activation of persistent sodium conductance [86]. Maximal facilitation (+127%, Fig 4C) appears larger than in LFP recordings (Fig 3) but this does not necessarily imply a difference between intracellular and extracellular recording: as previously reported [84], STP differed widely between cells and the average based on our limited sample of intracellular recordings was unlikely to match the average based on hundreds or thousands of cells simultaneously recorded in the LFPs.

### Short-term plasticity model

The experimental data suggested that STP at the LOT-layer Ia connection involves the interaction of facilitatory and depressant mechanisms with different temporal profiles, and moreover, that their relative contributions depended on extracellular calcium concentration. To achieve a quantitative description of these mechanisms, we used a model of STP adapted from models described in previous studies [53, 81, 82]. In order to satisfactorily fit the data, our model required 3 mechanisms: one facilitation and two depressions distinguished by their time constants of recovery. When experimental data were obtained with both 1.1 mM and 2.2 mM, the model was fitted on both datasets at once, with *E* as a shared parameter.

As highlighted in Fig 2C, the STP model reproduced the experimental data quite well (1.1 mM calcium: red line; 2.2 mM calcium: green line). The model suggests that, in this example, the dynamics of STP in 1.1 mM calcium can be accounted for by the presence of 3 phenomena: a facilitation mechanism with a recovery time constant of 92 msec, and two depression mechanisms that recovered with time constants of 18 and 87 msec. Yet the slowest depression impinged on only 2% of the available reserve (*k* = 0.98). Thus, response enhancement up to 25 Hz can be accounted for by the presence of short-term facilitation. Response enhancement was less marked at higher frequencies as a consequence of the recruitment of the short-term depression mechanism with a short time constant of recovery, which counteracted facilitation.

The model fitted to the data obtained in 2.2 mM calcium suggests the following changes to explain the differences observed between the two conditions: an 89% increase in resource utilization for the first response (*U*) accounts for the increased response amplitude in 2.2 mM calcium (*U* = 0.666 in 2.2 mM calcium *vs*. *U* = 0.353 in 1.1 mM calcium). The time constant of facilitation appears to be longer (223 msec). Yet, *k* decreased (*k* = 0.909), revealing a more important utilization of the slow depression mechanism than in control condition, that partially counteracted facilitation even at low frequency. Therefore the greater depression observed with the increase of calcium concentration can be explained by the increase of *U*–leading to stronger resource utilization–while appearance of depression for frequency lower than in 1.1 mM calcium results from the change in the value of *k*.

**Fig 5:**
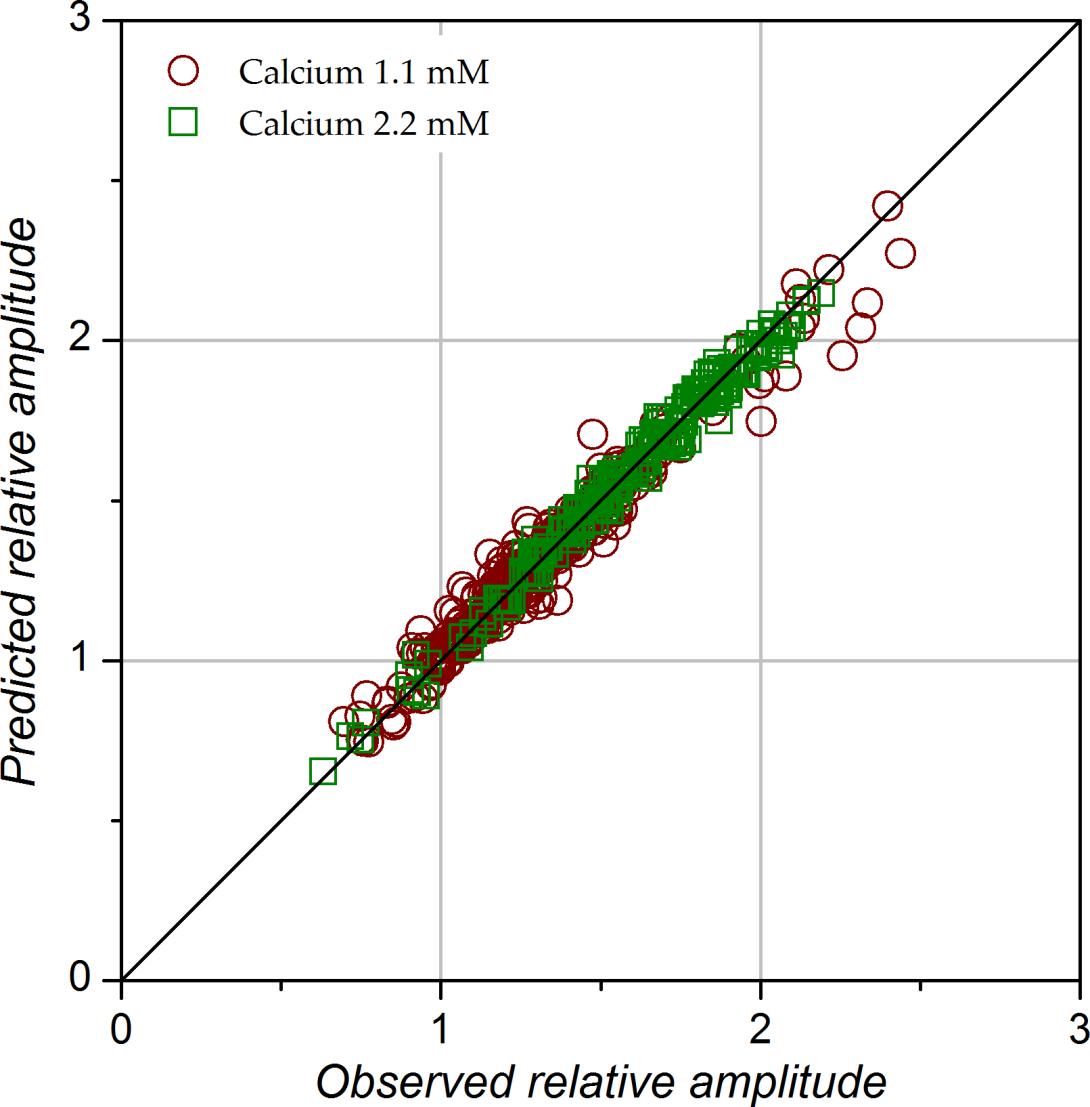
Scatterplot of RA values predicted by the model vs. observed RA values (n = 715 data points). Diagonal represents line of equality. The r^2^ of the linear correlation between predicted and measured values was 0.962 in 1.1 mM calcium and 0.986 in 2.2 mM calcium.

The population data for the relative amplitudes predicted by the model are represented by magenta circles (1.1 mM calcium) and blue squares (2.2 mM calcium) in Fig 3. Overall, the predicted values overlap well with the experimental data. Ability of the model to properly fit the data is further illustrated in Fig 5, which shows that the predicted response amplitudes are highly correlated with the amplitudes actually measured (correlation r^2^ = 0.962 in 1.1 mM calcium and 0.986 in 2.2 mM calcium).

The overall quality of the model fit was estimated by the RMSE. At the population level the mean RMSE was 0.047 [0.036–0.058] (n = 17). We tested other variants of the model. The first variant was a model with only one depression mechanism (as in: [81, 82]). The RMSE values returned by this first variant were significantly higher (*P* = 0.003, paired t-test) by +45% [+11%—+79%] on average in comparison to the initial model. The second variant was one in which parameter *E* was allowed to vary in the different calcium concentration conditions. For the 8 experiments in which the 2 calcium concentrations were tested, the mean RMSE was significantly (*P* = 0.008) lower (mean difference: -15% [-24%—-6%]), but this had the inconvenience of assuming that *E*, the total synaptic resource, varies with calcium concentration. The third variant was a model in which, in addition to *E*, the time constants of recovery from depression and facilitation were shared between the two calcium concentrations. This third variant performed less well on average: the RMSE was significantly (*P* = 0.03) increased by +32% [+11%—+53%] on average in comparison to the original model.

**Fig 6:**
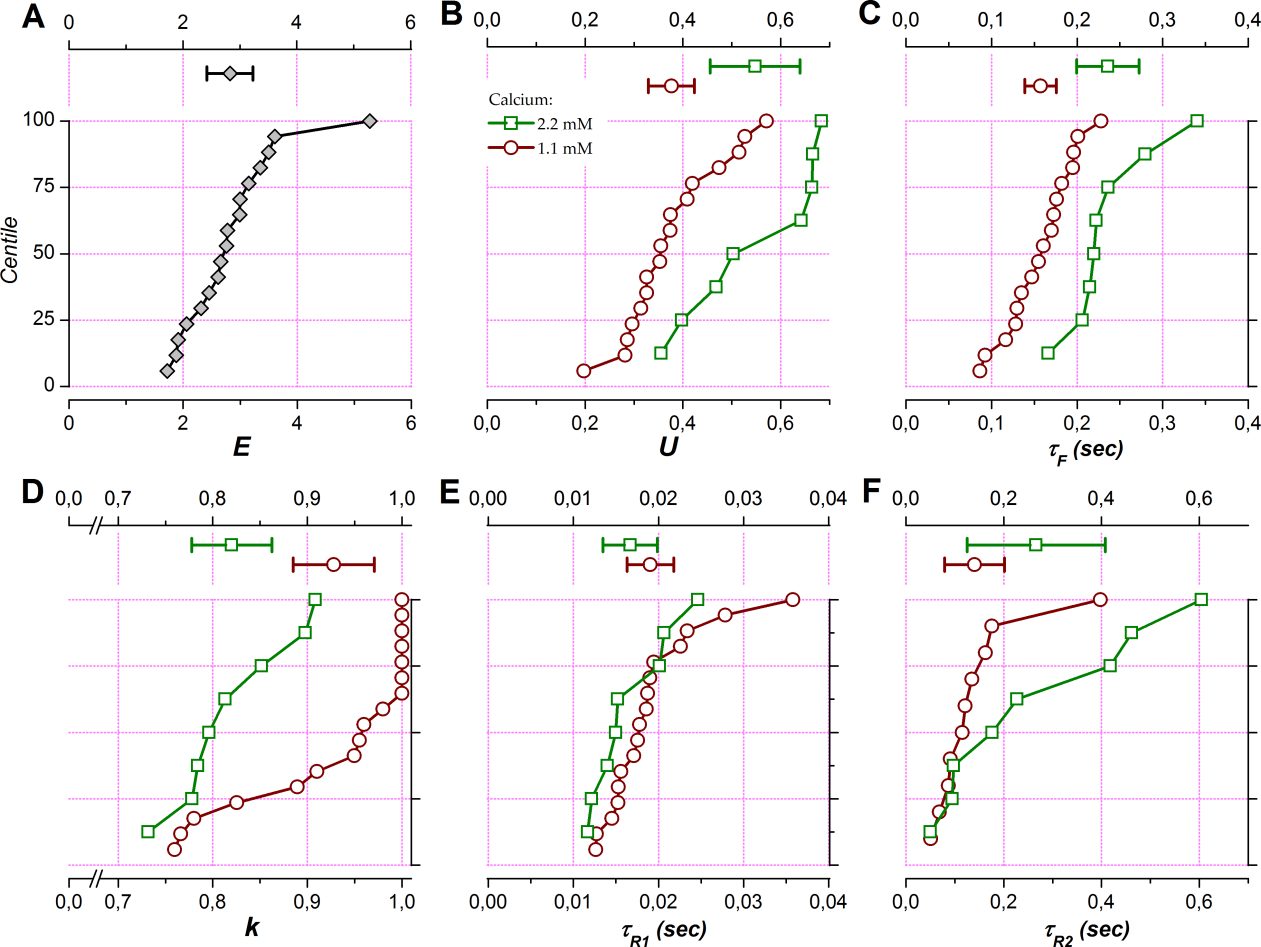
Short-term plasticity parameters at population levels with 1.1 and 2.2 mM calcium. Parameters optimized to fit the observed STP data are summarized as means and 95% confidence intervals (upper part in each panel) and as cumulative distributions (centile plots, lower part in each panel) for data obtained in 1.1 mM (red circles) and 2.2 mM calcium (green squares). The dashed line at 50% in the centile plots indicates the median of the distributions and lines at 25% and 75% delineate the interquartile range. *A*: *E*, efficacy. *B*: *U*, utilization of efficacy on a single stimulation at rest (fully recovered and non-facilitating). *C*: *τ*_*F*_, time constant of facilitation. *D*: *k*, coefficient defining the partition of synaptic resources in two subgroups subjected to different time courses of recovery from depression. *E*: *τ*_*R1*_, time constant of recovery for fast depression. *F*: *τ*_*R2*_, recovery time constant of recovery for slow depression.

**Fig 7:**
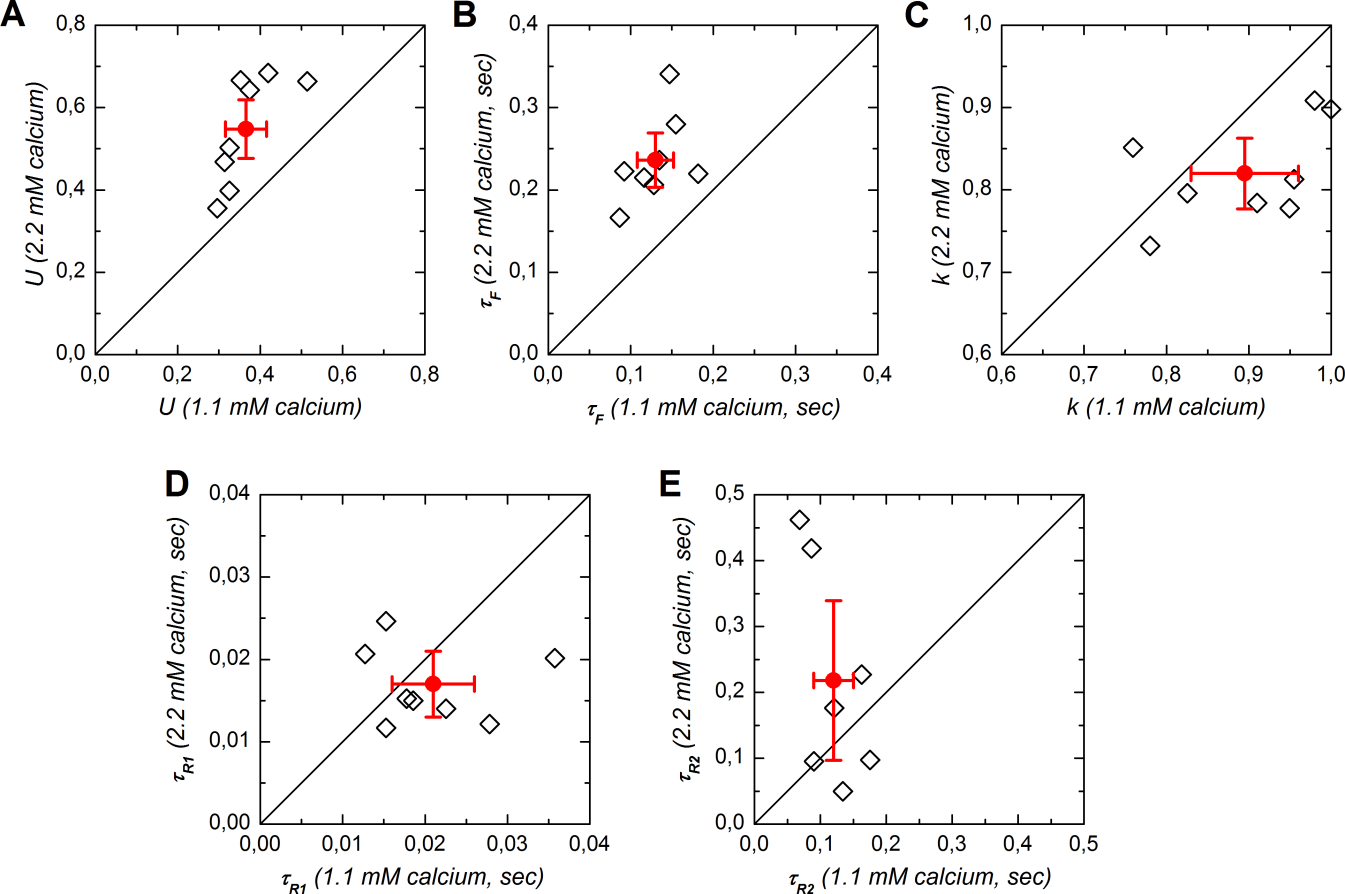
Scatter plot of short-term plasticity parameters for experiments performed with both 1.1 and 2.2 mM calcium (n = 8). *A*: *U*, utilization of efficacy on a single stimulation at rest (fully recovered and non-facilitating). *B*: *τ*_*F*_, time constant of facilitation. *C*: *k*, partition of synaptic resources. *D*: *τ*_*R1*_, time constant of recovery for fast depression. *E*: *τ*_*R2*_, recovery time constant for slow depression. Diagonal lines represent equality lines. Open symbols correspond to individual experiments, plain symbols represent the mean for the subsample of experiments in which STP was tested in both 1.1 and 2.2 mM calcium, associated bars correspond to the 95% confidence intervals.

Distribution of model parameter values are presented in Fig 6 while Fig 7 presents scatter plots for each parameter in 1.1 vs. 2.2 mM Ca^++^.

The parameter *E* corresponds to the maximum potential response, that is, a ceiling level for the response amplitude given the experimental conditions of the study. The mean value returned by the model was 2.825 [2.422–3.228] (Fig 6A). In other words, *E* represents, on average, 2.8 times the response amplitude at the first stimulation of a train in 1.1 mM calcium.

The parameter *U* allows defining the first level of *u* and corresponds to the fraction of *E* that is used at the first stimulation of a train. *U* increased with calcium concentration: *U* in 1.1 mM Ca^++^ was distributed from 0.198 to 0.571, with a mean of 0.377 [0.329–0.424] (Fig 6B). *U* was larger (+45%) in 2.2 mM Ca^++^, with a mean at 0.548 [0.456–0.639]. Fig 7A shows the effect of increasing calcium concentration in a pairwise fashion. The increase of *U* was observed in all cases and was highly significant (*P* = 0.001, paired t-test).

Likewise, the time constant of facilitation was strongly affected by increasing Ca^++^ (Fig 6C). The mean value of *τ*_*F*_ in 1.1 mM Ca^++^ was 157 msec [0.139–0.176] and appeared to be shorter than the mean value obtained in 2.2 mM Ca^++^, which was 236 msec [0.199–0.272]. When examined in a pairwise fashion we again notice a systematic and highly significant (*P* = 0.0003) increase of *τ*_*F*_ in 2.2 mM Ca^++^ (Fig 7B). The mean increase was +86% [+59%—+112%].

The parameter *k* determines the allocation of the synaptic resources into two subgroups, one that shows a fast and the other a slow recovery from depression. A value of 1 corresponds to cases where the slow depression mechanism was not required for fitting the data. This was the case in 6 of the 9 experiments where tests were made with 1.1 mM calcium only. On the other hand, *k*<1 in most of the experiments (7/8) where both 1.1 and 2.2 mM calcium were tested (Figs 6D and 7C). Yet in these cases, *k* was on average higher in 1.1 mM calcium than in 2.2 mM calcium (*P* = 0.038, Fig 7C). On average, the value of k was 0.82 [0.78–0.86] in 2.2 mM Ca^++^, meaning that about 20% of the synaptic resource underwent a process of slow recovery from depression. This proportion was less in 1.1 mM Ca^++^: for the whole sample, the mean value of *k* was 0.93 [0.88–0.97] and it was 0.89 [0.83–0.96] for the subsample of experiments in which both 1.1 and 2.2 mM Ca^++^ were used.

Since *k* >> 0 in all cases, the depression mechanism with fast recovery was systematically observed in both 1.1 and 2.2 mM Ca^++^. The recovery time constant for this mechanism, *τ*_*R1*_, was independent from calcium concentration (*P* = 0.3, Fig 7D). *τ*_*R1*_ averaged 19 msec [16–22 msec] in 1.1 mM Ca^++^ and 17 msec [13–20 msec] in 2.2 mM Ca^++^ (Fig 6E).

The depression mechanism with slow recovery displayed a recovery time constant, *τ*_*R2*_, that averaged 140 msec [79–202 msec] in 1.1 mM Ca^++^ and 266 msec [125–407 msec] in 2.2 mM Ca^++^ (Fig 6F). Despite a trend for being slightly longer in 2.2 mM Ca^++^, the *τ*_*R2*_ values did not differ significantly between the two calcium concentrations (*P* = 0.2, Fig 7E). The slow depression recovery time constant was of the same order of magnitude as the facilitation time constant but there was no correlation between *τ*_*F*_ and *τ*_*R2*_ (r^2^ = 0.26 and *P* = 0.5 in 1.1 mM Ca^++^, r^2^ = 0.45 and *P* = 0.3 in 2.2 mM Ca^++^, not illustrated).

**Fig 8:**
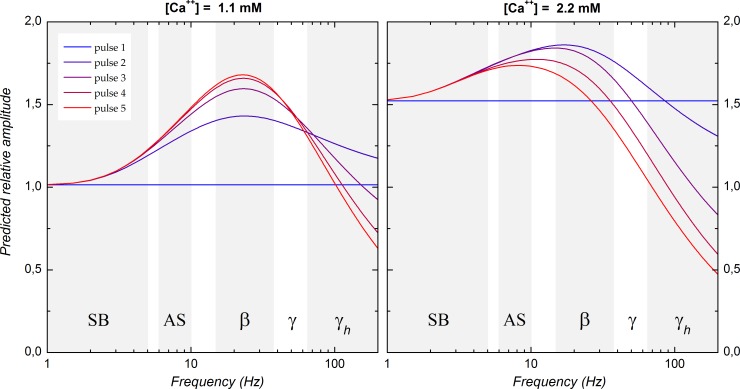
Relative response amplitude as a function of stimulation frequency reconstructed from short-term plasticity model parameters in 1.1 mM calcium (left) and 2.2 mM calcium (right). The simulation was computed using the mean of the parameter values after excluding one extreme value on each side of the distributions to limit possible influence of outliers. The frequency range has been subdivided into frequency bands typically observed in rodent olfactory bulb: SB, slow breathing (1–5 Hz); AS, active sniffing (6–10 Hz); β, beta band (15–37.5 Hz); γ, gamma band (37.5–65 Hz); γ_h_, high gamma band (>65 Hz). The alpha band (8–12 Hz) is not represented as it partially overlaps with the AS band.

The parameters returned by the model allowed reconstructing STP over a continuum of stimulation frequency. Fig 8 displays the predicted relative response amplitudes as a function of pulse ordinal number and stimulation frequency. In 1.1 mM calcium, the simulated RA reached a maximum that remained at 23 Hz with all stimulating pulses. The dynamic range increased from +41% with the second pulse to +65% with the fifth pulse, in close agreement with the experimental data (+35% and +58%, respectively, at 25 Hz). The range of frequencies over which response amplitude was more than half the maximal amplitude obtained for each pulse was quite broad with the second pulse (6–130 Hz) but narrowed with the following pulses to be restricted to the frequencies corresponding to active sniffing and to the beta and gamma band (7–76 Hz with the second pulse, 7–60 Hz with the fifth pulse).

In comparison to that observed in 1.1 mM, the dynamic range was reduced in 2.2 mM calcium, with a maximal enhancement at +22% with the second pulse and at +14% only with the fifth pulse. The frequency at which the maximum RA was reached was lower with the second pulse (17 Hz) and furthermore, it progressively drifted toward lower frequency with the following stimulation pulses (8 Hz with the fifth pulse). The half maximal amplitude frequency range drifted in parallel (5–50 Hz with the second pulse, 3–19 Hz with the fifth pulse). Finally, except for second pulse response, RA decreased below first pulse amplitude for frequencies within the gamma range (third pulse) and within the beta band (fifth pulse) whereas stimulation frequencies >100 Hz would be required to induce such reduction in 1.1 mM calcium.

Altogether these data show that the features of STP at the LOT-layer Ia synapse endows piriform cortex with the ability to amplify its afferent olfactory inputs. Yet using a “classical” extracellular calcium concentration (2.2 mM) resulted in underestimating both the amplification and the frequencies over which amplification took place. Using an *in vivo*-like ACSF revealed a larger amplification of synaptic responses especially when stimulation frequency straddled the range reported to be associated with odor-induced oscillations in the olfactory bulb.

## Discussion

Our experimental data showed that, provided extracellular calcium concentration corresponds to that measured *in vivo*, response dynamics at the LOT-layer Ia synapse is characterized by a considerable response enhancement. This enhancement was maximal in the beta frequency range, although it also impinged on the gamma frequency range and on the range of frequencies associated with active sniffing. The STP model fitted to the data indicated that this enhancement was essentially achieved through the interaction of 3 mechanisms: a facilitation mechanism with a recovery time constant of 157 msec on average, that resulted in a progressive increase in response amplitude when stimulation frequency was > about 2 Hz, a fast depression mechanism with a recovery time constant of 19 msec that curtailed facilitation when stimulation frequency was > 23 Hz, and, in some cases, a depression mechanism with slower recovery. Increasing calcium concentration to 2.2 mM unsurprisingly increased the amplitude of responses evoked at low frequency but it also reduced the dynamic range of response enhancement. Furthermore, depending on pulse ordinal number, the frequency at which enhancement was maximal drifted from the low beta band to the alpha band. Finally, at stimulation frequency >30–50 Hz, depression took over to the extent that response amplitude could be similar to that obtained in 1.1 mM calcium. The STP model provided three explanations for the changes induced by increasing calcium concentration: the longer time constant of facilitation accounted for part of the shift of enhancement toward lower frequencies; the slow depression mechanism, more prominent than in 1.1 mM calcium, also contributed to the shift of response enhancement toward lower frequencies; finally larger depletion of synaptic resources with the first pulse resulted in reduced reserve availability, in turn leading to stronger depression at high frequency.

### Effect of calcium on response amplitude at low stimulation frequency

That postsynaptic response amplitude depends on extracellular calcium concentration is a well established fact that has been reported in numerous studies. Quantitative studies exploring this relationship initially examined the initial portion of the calcium concentration vs. response amplitude curve that can be linearized in logarithmic representation, while later studies relied on fitting the whole relationship with, among others, Hill equation as in the present study (Fig 1). These studies all demonstrated a nonlinear relationship between extracellular calcium concentration and response amplitude, with an exponent between 2 and 5 [87–95]. An exponent value of 2.5 has previously been reported in the piriform cortex [90], quite close to the Hill coefficient value of 3 reported here. A Hill coefficient of 3 suggests the cooperative action of three calcium ions in neurotransmitter release at the LOT-layer Ia synapse. Similar degree of cooperativity has been reported in other preparations [88, 93, 95]. The nonlinearity in the relationship between calcium concentration and response amplitude leads to the presence of a saturation level. This saturation level varies between synapse types, being ≈10 mM in the neuromuscular junction [88] and ≈5 mM in hippocampus [93]. Interestingly, in neocortex Rozov et al. (2001) [94] found that the saturation level depended on the identity of the postsynaptic neurons rather than that of the presynaptic neurons: ≈2 or ≈5 mM depending on whether the neurons postsynaptic to pyramidal cells were multipolar or bitufted interneurons. Our data also revealed the presence of a saturation of response amplitude with a calcium concentration of ≈5 mM in piriform cortex.

### Comparison with previous short-term plasticity studies in piriform cortex

Short-term synaptic plasticity in piriform cortex has been examined in a number of studies, both *in vivo* and *in vitro*. Without exception all the studies relying on paired-pulse protocols revealed paired-pulse facilitation at the LOT-layer Ia synapse [84, 96–101]. The interpulse interval eliciting maximal facilitation varied from one study to another but most studies reported optimal intervals falling within the beta frequency range. Nevertheless, approaches based on paired-pulse protocols do not allow examining the whole dynamics of STP. For example, in our case the paired-pulse ratio indicated a maximal facilitation in the low beta range in 2.2 mM calcium, yet facilitation appeared to drift toward lower frequencies with additional stimuli (Fig 8). Fewer studies examined STP with trains of pulses [82, 97, 100, 102–104]. Qualitatively, our results conform with those obtained in these previous studies, although discrepancies can be noticed that may in part be attributed to differences in stimulation protocols, in animal's developmental stage, and, for *in vitro* studies, in ionic composition of the ACSF (see below). In relation to these issues, it is noteworthy that our results (Figs 2 and 3) are very close to those obtained by Richards (1972) [97] under experimental conditions very similar to ours. Thus, typically, at the LOT-layer Ia synapse, response enhancement is observed over a range of frequencies that includes the alpha, the beta and, eventually, the gamma bands; at high stimulation frequency, recruitment of depression mechanisms tend to counteract response enhancement.

### The phenomenology explained by the model

The phenomenology of STP at the LOT-layer Ia synapse is quite well established. We wanted to go further by unraveling the underlying mechanisms. For this purpose we fitted our experimental data with an STP model (adapted from: [53, 81, 82]). Optimal fit of the data required three distinct mechanisms: a facilitation mechanism and two depression mechanisms. The depression mechanisms operated on two time scales. Interestingly, the contribution of the slowest of the two depression mechanisms depended on extracellular calcium concentration. Extracellular calcium concentration also affected the time course of facilitation.

The time course of facilitation we report here is comparable to that reported at several other synapses (e. g., [105–110]). Several models of STF have been proposed (reviewed in: [29, 111]): the classical “residual calcium hypothesis” [112], which posits that accumulation of free calcium in the presynaptic terminals facilitates subsequent release of neurotransmitters; presence of high-affinity calcium binding sites with slow recovery, that would either cooperate with the main release sensor or that would facilitate presynaptic calcium current [95, 107, 113–116]; and saturation of endogenous calcium buffers [94, 117].

We found that the time constant of recovery from facilitation was longer with higher calcium concentration (Figs 6 and 7). Although it is well established that changing extracellular calcium concentration determines that amplitude of facilitation, we are unaware of studies demonstrating that such manipulation also affects the time course of facilitation. This calcium dependency suggests that the free residual calcium model is not sufficient to explain facilitation: extrusion of free calcium should display a single time constant independently of intracellular calcium concentration. Jackson and Redman (2003) [118] showed that the time constant of calcium concentration decay in synaptic terminals, *τ*, can associate two mechanisms, summarized by the equation: *τ* = *τ*_*0*_ × (1+*κ*_*E*_), where *τ*_*0*_ corresponds to the time constant of the calcium extrusion mechanism and *κ*_*E*_ to the calcium binding capacity of an endogenous calcium binding protein. An increase in *τ*_*f*_ would readily be explained if *κ*_*E*_ increases with extracellular calcium concentration.

Our results also suggest that two different depression mechanisms operate at the LOT-layer Ia synapse in our experimental conditions (Figs 6 and 7): the first one, observed in all cases, was characterized by a fast recovery time constant (<20 msec on average); the second one, whose involvement depended on extracellular calcium concentration, displayed a slower recovery time constant (100–200 msec). As these two mechanisms were revealed with relatively few stimuli, we cannot exclude additional depression mechanisms that would be revealed with longer stimulus trains. Actually, an additional depression mechanism recovering within 100 sec has been evidenced in piriform cortex with stimuli consisting in trains of hundreds of pulses [97, 102].

As for facilitation, several models have been proposed for explaining STD. Few of these mechanisms are postsynaptic and most are presynaptic. As these different mechanisms are associated with different time courses, several may operate at a given synapse.

One postsynaptic mechanism of STD, namely AMPA receptor desensitization, has been proposed to operate at some central synapses [119, 120]. Interestingly, the time constant of recovery for this mechanism is quite fast [120–122] and appears to be similar to the time constant of recovery for the fast depression mechanism we report for the LOT-layer Ia synapse.

More traditionally, STD has been attributed to presynaptic mechanisms (reviewed in: [29, 111, 123, 124]. Among these, a fast mechanism recovering also in a few tens of msec has been reported at various synapses (e. g., [125–128]). One possible explanation for this short-lasting STD is a refractoriness of the release sites: once the immediately releasable vesicle pool has been exocytosed, release sites would be unavailable for a second release. Short-lasting inactivation of calcium currents has been proposed as an alternative mechanism [126].

Another presynaptic mechanism of STD corresponds to the emptying of the readily releasable pool of synaptic vesicles. This well documented mechanism has been demonstrated mostly in protocols based on large number of stimuli (e. g., [129–131]). Furthermore, recovery of the readily releasable pool appears to be a slow process characterized by time constants of seconds to minutes (e. g., [102, 132–134]). Our stimulation protocol was based on few stimulating pulses, and the recovery from depression was complete in much less than one second. This suggests that exhaustion of the readily releasable pool of vesicles was not the mechanism involved in our data.

In between fast and slow recovery mechanisms, depression mechanisms recovering with a time constant of the order of a few hundred msec, as our second depression mechanism, have been reported in multiple structures (e. g., [82, 108, 121, 135–137]). In our experiments we found that the strength of this type of depression increased with higher calcium concentration. Comparable calcium dependency has been reported at other synapses. In particular, at the climbing fiber to Purkinje cell synapse, Dittman and Regehr (1998) [135] demonstrated the presence of a depression mechanism, nearly lacking in 1 mM extracellular calcium, whose recovery accelerated in proportion of the extracellular calcium concentration. A calcium-dependent recovery process has also been observed in neocortex [136]. Beside, studies demonstrated that inactivation of voltage-dependent calcium channels can contribute to STD (reviewed in: [95, 111, 124]). As for facilitation, calcium-dependent STD could implicate calcium binding proteins, that could in turn regulate various processes such as voltage-dependent calcium channel inactivation or recovery of the immediately releasable vesicle pool [95, 114, 115, 135].

### Effects of animal age and of extracellular calcium on short-term plasticity

Developmental stage, ionic concentrations and temperature are important variables in STP. We wished to examine STP in experimental conditions that were as close as possible to those prevailing in the adult brain *in vivo*. For this purpose we used brain slices, maintained at near physiological temperature, from adult mice and we took care to use extracellular ionic concentrations as close as possible to those reported in the interstitial fluid *in vivo* (see Methods).

A number of studies examining STP have been performed at room temperature, yet STP mechanisms may be differently affected by temperature. For example, Klyachko and Stevens (2006) [109] revealed profound effect of temperature on STP in hippocampus: depression dominated at room temperature but facilitation and augmentation prevailed when temperature ≥33°C.

It is also now well established that STP is strongly impinged by the developmental stage. For example, studies in neocortex demonstrated that STD, which prevails in immature animals, is reduced in adults while STF becomes more prominent [108, 138–143]. These developmental changes may be the consequence of an increase in endogenous adenosine concentration [137]. Developmental changes in STP have been reported also in a number of other structures, for example in the hippocampus (e. g., [144, 145]) and at the calyx of Held synapse [146].

Yet, even with adult animals, studies examining STP *in vivo* [110, 147–151] typically reported more facilitation and/or less depression than in analogue structures studied *in vitro*. Part of the difference might be explained by the presence of spontaneous activity that results in a steady state depression *in vivo* [148, 150, 152]. Another difference between *in vivo* and classical *in vitro* studies is calcium concentration: 1–1.2 mM *in vivo* vs. 2–2.5 mM in most *in vitro* studies. One reason initially invoked for justifying the use of high calcium concentration *in vitro* was that it improved the stability of intracellular recordings [153]. Hence a number of studies also demonstrated strong effects of calcium concentration on STP. In particular studies comparing STP in “classical” *in vitro* calcium concentration and in *in vivo*-like calcium concentration showed larger facilitation and/or reduced depression with the later [109, 110, 135, 150, 151, 154–156].

Our study is a further demonstration of the importance of calcium concentration in STP, here in piriform cortex. Noticeably, had we stuck to the traditional approach of using young animals and high calcium concentration, we would have missed the large response enhancement in response to stimuli delivered in the beta frequency. Linking short-term plasticity in piriform cortex to rhythmic activity in the olfactory bulb would have then been more tentative.

## Conclusion

Our results indicate that the interplay of three short-term plasticity mechanisms results in a frequency dependent enhancement of postsynaptic response amplitude at the LOT-layer Ia synapse in piriform cortex. In the *in vivo*-like ACSF the maximal enhancement was observed in the beta frequency range but was still large in the gamma frequency range (Fig 8). Beta and gamma oscillations are typically associated with olfactory stimulation in the olfactory bulb [13, 35–49]. Thus, in contrast to the hypothesis that oscillations are simply an epiphenomenon of circuit architecture, our results suggest that oscillations in the olfactory bulb have a functional impact on information transfer in downstream structures. More generally, beta and gamma oscillations are observed in a number of cortical and subcortical structures and are associated with sensory perception and with various cognitive tasks. It remains to be determined whether postsynaptic response enhancement in response to afferent stimulation at beta and gamma frequencies also takes place in these structures.

## Supporting information

S1 Dataset Individual data (n-wave amplitude as a function of calcium concentration, stimulation frequency and pulse ordinal number) are made available in the supporting information file S1_dataset.xls. (XLS)
